# Double-Network Hydrogels of Corn Fiber Gum and Soy Protein Isolate: Effect of Biopolymer Constituents and pH Values on Textural Properties and Microstructures

**DOI:** 10.3390/foods10020356

**Published:** 2021-02-07

**Authors:** Jinxin Yan, Xin Jia, Wenjia Yan, Lijun Yin

**Affiliations:** Beijing Key Laboratory of Functional Food from Plant Resources, College of Food Science and Nutritional Engineering, China Agricultural University, No. 17 Qinghua East Road, Haidian, Beijing 100083, China; yanjinxin@cau.edu.cn (J.Y.); xinjia@cau.edu.cn (X.J.); sevenyan@cau.edu.cn (W.Y.)

**Keywords:** corn fiber gum, soy protein isolate, double-network hydrogel, biopolymer constituent, pH value

## Abstract

Corn fiber gum (CFG) -soy protein isolate (SPI) double-network (DN) hydrogels were fabricated using laccase and a heat treatment process, in which CFG solution formed the first gel network via laccase oxidation, while SPI formed the second network through heating, as described in our previous research. The aim of this study was to investigate the influences of CFG/SPI constituents (CFG concentration 0–3%, *w*/*v*; SPI concentration 8–10%, *w*/*v*) and pH values (5.0–7.5) on the textural properties, microstructures and water-holding capacities (WHC) of the CFG-SPI DN hydrogels. Confocal Laser Scanning Microscopy (CLSM) results showed an apparent phase separation when the CFG concentration was above 1% (*w*/*v*). The textural characteristics and WHC of most DN hydrogels were enhanced with increasing concentrations of CFG and SPI. Scanning Electron Microscopy (SEM) observations revealed that the microstructures of DN hydrogels were converted from coarse and irregular to smooth and ordered as pH values increased from 5.0 to 7.5. Excellent textural properties and WHC were observed at pH 7.0. This study developed various CFG-SPI DN hydrogels with diverse textures and structures, governed by the concentrations of protein/polysaccharide and pH values, and also contributes to the understanding of gum–protein interactions in DN hydrogels obtained under different conditions.

## 1. Introduction

Hydrogels are three-dimensional network structures comprising one or more polymers with covalent or noncovalent interactions between chains that are capable of retaining significant quantities of water or biological fluids without dissolving [1,2]. Hydrogels have been used as delivery vehicles for various biological materials, such as probiotics and bioactive compounds, to protect these functional substances from being degraded by digestive enzymes and release them in target regions of the gastrointestinal system [1]. Research on the mechanical and functional properties of food hydrogels is of great significance for the development of food products with different texture profiles that meet consumer demands [3]. Proteins and polysaccharides are the two most common biopolymer types used to construct food hydrogels due to their excellent gelling properties [4,5]. In recent years, many researchers have used combinations of a polysaccharide and a protein to fabricate mixed hydrogels to improve the mechanical properties and stability of individual biopolymer hydrogels [3,6,7]. However, in most current protein-polysaccharide mixed hydrogel systems, polysaccharides are mainly used as intermolecular fillers, which improve the characteristics of mixed gels through thickening, cosolubilization, ion complexation, and phase separation [8,9,10]. These types of mixed hydrogels are characterized by weak interactions and still belong to single-network hydrogels [4]. The improvement effect of gel strength and mechanical properties is limited. Therefore, double-network (DN) hydrogels were developed as a feasible strategy to overcome the defects of single-network gels, that is, using various gelation methods to induce proteins and polysaccharides to form complex multiple networks in one gel system; following this approach, the textural properties could be significantly improved [11]. DN hydrogel was first proposed by Gong et al. [12], who applied two different combinations of hydrophilic polymers (e.g., highly cross-linked polyelectrolyte PAMPS and neutral polymer PAMPS) to prepare double-network hydrogels with remarkably high mechanical strength by using a two-step method. The highly heterogeneous and complex entangled network structure were believed to be responsible for the extremely high strength and toughness of the DN hydrogels, compared with traditional, single-network hydrogels. Recently, Guo et al. [5] developed an edible gellan gum-SPI DN hydrogel using a sequential ionic-covalent gelation method, in which a gellan gum hydrogel network was formed as the first rigid skeleton through KCl induction, and a SPI hydrogel network was formed as the second ductile network through microbial transglutaminase catalysis. The appropriate combination of a rigid polysaccharide hydrogel network and a ductile protein hydrogel network in the DN hydrogel contributed to its excellent mechanical strength and toughness, as well as order and dense network structure [5]. Furthermore, previous studies have confirmed that these mixed biopolymer gels could possess enhanced mechanical and other functional properties, i.e., taste profiles and digestive breakdown [13,14]. The development of tailored DN hydrogels with specific mechanical properties, different from individual component hydrogels, provides new ideas for the preparation of edible hydrogels with various textures.

However, DN hydrogels are generally composed of more than one type of biopolymer, and thus, the polymer characteristics, as well as environmental factors, are crucial in the fabrication process [12,14,15]. Their mechanical properties and microstructures can be tuned by controlling various parameters, such as the concentrations of protein and polysaccharide, pH, ionic strength, and temperature. Hou et al. [3] demonstrated that the highly heterogeneous and hierarchical microstructure of a sugar beet pectin-soy glycinin DN gel was attributed to the phase separation behavior of the two biopolymers at different length scales; as the polysaccharide concentration increased, the phase separation and heterogeneity of the network enhanced, which, in turn, lead to greater mechanical toughness of the DN gel. The occurrence of phase separation behavior is very common in protein/polysaccharide mixed gel systems due to the thermodynamic incompatibility of biopolymers; as such, the resultant gel structure and properties are usually governed by competition between phase separation and gelation [16,17]. In multiple food products, the composition and concentration of each component play decisive roles in the phase separation behavior of mixed systems. In addition, previous studies have shown that pH has a great influence on the rheological properties and microscopic phase behavior of polysaccharide and protein mixed systems [15,18]. Carrageenan can change the denaturation and gelling rates of soy protein through its synergistic effect at pH values lower than the protein isoelectric point, as reported by Zhou et al. [19]. Some studies have verified that the change of the system pH is very significant in the regulation of the microstructure of either protein gels or polysaccharide gels [20,21]. Therefore, it is feasible to obtain ideal DN hydrogels with different length scales and mechanical properties by manipulating the concentration of protein or polysaccharide of the mixed system and environmental conditions (such as pH, etc.). This knowledge opens the way for the food industry to produce hydrogel-based food products with various texture profiles.

In the present study, corn fiber gum and soy protein isolate were used as the gel matrix. Corn fiber gum (CFG) is a kind of hemicellulose derived from corn bran, which is constituted by a linear backbone of β-1,4-linked xylose units containing α-L-arabinofuranosyl substituents attached through O-2 and/or O-3 [22,23]. The presence of ferulic acids covalently attached to the side chain arabinose of CFG confers excellent gelling ability that allows CFG to form a gel through the oxidative crosslinking of ferulic acids under the action of laccase or peroxidase [24]. Most of the current research focuses on its extraction, structural composition and emulsifying properties [25,26]; few studies have examined its gelling property, and even fewer the fabrication of DN hydrogels using CFG and other polymers. Soy protein isolate (SPI), which can form hydrogel either by heat-set or cold-set mechanisms, is one of the most popular gelling agents in the food industry. It mainly consists of glycinin (11S) and β-conglycinin (7S), which account for approximately 70% of its total protein content [20,27]. Nevertheless, the gelling capacity of SPI, which is strongly influenced by pH and ionic strength, is poor. Our previous work constructed a method of forming DN hydrogels using CFG and SPI as raw materials through laccase and heat treatment [11]. The results showed that the CFG-SPI DN hydrogels combined the dual advantages of single-polysaccharide or protein hydrogels, and exhibited outstanding properties such as increased hardness compared to SPI hydrogels and better deformation ability compared to CFG hydrogels [11]. However, the effects of CFG/SPI constituents and pH on the properties of CFG-SPI DN hydrogels are not clear. Therefore, the aim of this study is to clarify the influence of these factors on the mechanical properties and microstructures of DN hydrogels, and to obtain edible gels with diverse structures and characteristics, which will be of great significance for the design of foods with excellent texture and taste.

## 2. Materials and Methods

### 2.1. Materials

Wet milled corn fiber was obtained from local corn kernel processors. Soy protein isolate (SPI) was kindly provided by Fuji oil Co.Ltd Beijing Branch. Laccase from Trametes versicolor (E.C.1.10.3.2) was purchased from Sigma-Aldrich (St. Louis, MO, USA). Heat-resistant α-amylase was purchased from Aladdin Industrial Corporation (Shanghai, China). All other reagents were of analytical grade.

### 2.2. Isolation of CFG

The extraction process was based on our previously published article [11]. Briefly, wet milled corn fiber that was ground to 40-mesh was firstly added to mechanically stirred n-hexane (The ratio of corn fiber to hexane is 1:7 (*w*/*v*)) for 2 h in order to remove oil from the fiber. Subsequently, the deoiled fiber (100 g) was resuspended into 800 mL water and boiled for 1 h with mechanical stirring to fully gelatinize starch. Termamyl-amylase was added to hydrolyze and remove starch. CFG was isolated from the deoiled and destarched corn fiber (100 g) by mechanical stirring with 0.25 N sodium hydroxide solution (equal amount of each) per gram of fiber at 25 °C in the dark for 4 h. The resulting mixture was centrifuged at 7400× *g* for 15 min, and the supernatant was separated from the residue by decantation. The pH of the supernatant was then adjusted to 4.0–4.5 using concentrated HCl. The acidic mixture was centrifuged at 7400× *g* for 15 min to separate the precipitate (hemicellulose A). Four volumes of absolute ethyl alcohol were added to the collected supernatant to precipitate CFG, and finally, the precipitate was lyophilized to obtain dry CFG powder.

### 2.3. Preparation of CFG-SPI DN Hydrogels 

SPI powder was dispersed in deionized water by stirring for 4 h. The mixture was then stored at 4 °C overnight to obtain SPI stock solutions. Different concentrations of CFG stock solutions were prepared using a similar dissolving procedure. The resulting CFG stock solutions were then added slowly to the fully hydrated SPI solution with stirring at room temperature until fully dissolved. The final concentrations of CFG and SPI in the mixed dispersions were 0.00, 0.25, 0.50, 1.00, 2.00, 3.00%, (*w*/*v*) and 8.00, 10.00, 13.00%, (*w*/*v*), respectively. The pH of all mixed solutions was about 7.52–7.54. The CFG-SPI DN hydrogels were fabricated by using a two-step synthesis method induced by enzymes and heat treatment. Firstly, the solution of CFG-SPI mixture with the addition of laccase (1.670 nkat/mg CFG) was cultured at 25 °C for 2 h to generate the first hydrogel network of CFG. Secondly, the mixture was incubated in a water bath at 80 °C for 30 min and cooled to room temperature immediately by rinsing the outer walls of the hydrogel container with running water for the formation of the second hydrogel network of SPI. For tests of the effect of pH values on DN hydrogels, the final concentrations of CFG and SPI were 2 and 13% (*w*/*v*), respectively. Then, 2 N Hydrochloric acid and 2 N sodium hydroxide solution were used to adjust the pH of the CFG-SPI mixed solutions to 5.0, 5.5, 6.0, 6.5, 7.0, and 7.5, before laccase was added. 

### 2.4. Confocal Laser Scanning Microscope (CLSM) Observation 

To observe the spatial phase distribution of protein and polysaccharide in the DN hydrogel network CFG-SPI DN hydrogels at different concentrations of CFG and SPI, a Leica TCS SP5 Confocal Laser Scanning Microscope (Leica Microsystems Inc., Heidelberg, Germany) equipped with 40 times objective lens (40 × NA 0.85) was used. Rhodamine B (0.01% *w*/*v*, 1 μL/mL sample solution) was added to the CFG-SPI mixed dispersions that were prepared with a constant SPI concentration of 13% (*w*/*v*) and varying concentrations of CFG (0–3%, *w*/*v*) or a constant CFG concentration of 2% (*w*/*v*) and varying concentrations of SPI (8–13%, *w*/*v*) before the gelation steps. Laccase (1.670 nkat/mg CFG) was added to the above mixed sample solutions, which were then immediately transferred to glass slides and covered with a coverslip sealed by transparent nail oil. The slides were wrapped with tinfoil and maintained at 25 °C for 2 h, then at 80 °C in a water bath for 30 min and finally, cooled off quickly. The Rhodamine B was excited at 543 nm with a helium neon laser (He/Ne), and the emission fluorescence was recorded between 600–700 nm. The CLSM images were acquired in 1024 × 1024 pixel resolution; the green colored areas are the protein rich phases.

### 2.5. Textural Measurements

All hydrogel samples were formed in cylindrical beakers, giving the samples a fixed shape of 20 mm internal diameter × 20 mm height. Then, the freshly prepared hydrogels were stored at 4 °C for 12 h for subsequent analysis. Textual measurements of all hydrogels were done with a texture analyzer (CT3, Brookfield Engineering Laboratories, INC. Middleboro, MA, USA) equipped with a cylinder measuring probe with a diameter of 20.0 mm (P/20a). Samples were compressed to 30% of their initial height at a constant probe speed of 1.0 mm/s under two consecutive cycles of compression. All experiments were performed at room temperature, and parameters such as hardness, springiness and chewiness were calculated using the texture analyzer software. 

### 2.6. Water-Holding Capacity (WHC)

The WHCs of the hydrogels were determined based on the method described by Munialo et al. [28] with slight modifications. The hydrogels that had been stored at 4 °C for 12 h were taken out of the refrigerator and placed at room temperature for 2 h. Then, 3 g of each hydrogel sample was carefully placed in an ultrafiltration centrifuge tube (Millipore, Merck KGaA, Darmstadt, Germany) containing an inner tube with a strainer, and then centrifuged at 2900× *g* for 10 min at room temperature. In order to avoid small water droplets on the outer wall of the inner tube affecting the experimental results, the remaining water was carefully wiped off with filter paper. The water-holding capacity was calculated as a percentage as follows:

$$WHC=\frac{\left(W_{2}-W\right)}{\left(W_{1}-W\right)}\times 100\%$$

where *W* is the weight of the inner tube of the centrifuge tube, *W*_1_ is the initial total weight of the sample and inner tubes and *W*_2_ is the total weight of the sample and inner tubes after centrifugation.

### 2.7. Scanning Electron Microscopy (SEM)

The microstructure images of DN hydrogels prepared at different pH values were recorded by scanning electron microscopy (Quanta 200, FEI Company, Brno, Czech Republic). For microscopic analyses, the samples were frozen in liquid nitrogen for 5 min and then freeze-dried. The lyophilized samples were cut into small slices, 3 mm long and 3 mm wide, and attached to aluminum pin stubs using double-sided conductive carbon tabs. The small hydrogel slices coated with powdered gold on the surface were observed by SEI secondary electron imaging system at an accelerating voltage of 15 kV so as to obtain visual microstructure images. 

### 2.8. Statistical Analysis

All determinations were performed in triplicate. The analysis of variance (ANOVA) and Duncan’s means comparison test were evaluated at a significant difference level of α = 0.05. Data are expressed as the mean ± SD.

## 3. Results and Discussion

### 3.1. Effect of CFG/SPI Constituents on Properties of CFG-SPI DN Hydrogels

#### 3.1.1. Microscopic Phase Distribution Variation

**Figure 1:**
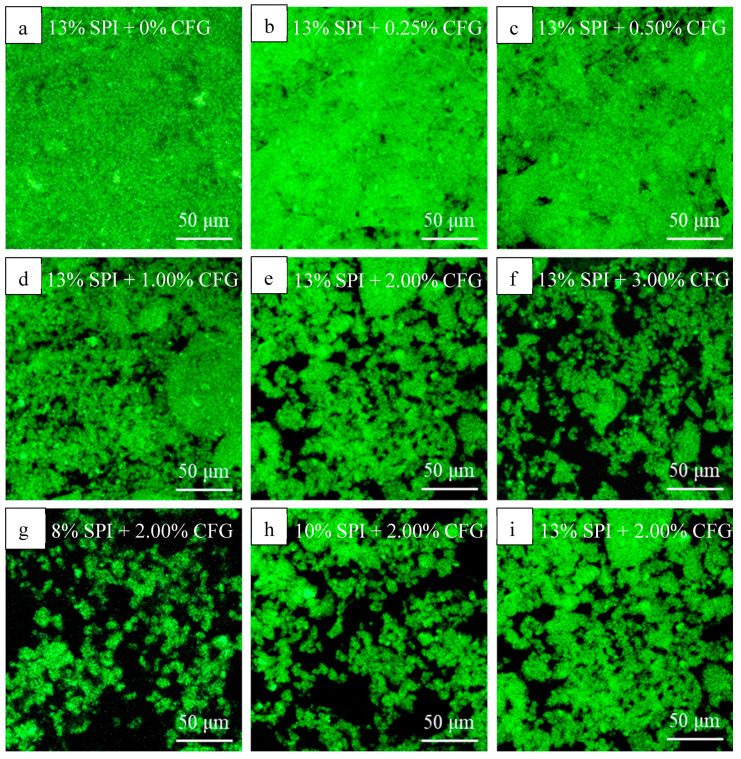
CLSM images of CFG-SPI DN hydrogels. 13% of SPI with various concentrations of CFG ((**a**) 0%; (**b**) 0.25%; (**c**) 0.5%; (**d**) 1%; (**e**) 2%; (**f**) 3%, (*w*/*v*)). 2% SBP with various concentrations of SPI ((**g**) 8%; (**h**) 10%; (**i**) 13%, (*w*/*v*)).

Figure 1a–f show the CLSM observations of the phase distribution of protein and polysaccharide in the DN hydrogels with the different CFG concentrations (0–3%, *w*/*v*) and a fixed concentration of SPI at 13% (*w*/*v*). The green areas were the SPI-rich phases stained with Rhodamine B and the black areas were the CFG-rich phases. SPI was evenly distributed in the system forming a single-network gel without CFG. When a small amount of CFG (0.25%, 0.50%, 1.00%) was added, a few black areas appeared among most of the green regions, which indicated that the SPI phase was the continuous phase in the mixed system, and that SPI hydrogel played a dominant role in the DN hydrogel system. As CFG content increased to 2% and 3%, the microstructure of the hydrogel changed from a continuous to a bicontinuous pattern. This could be explained by the microscopic phase separation behavior occurring in this polysaccharide-protein mixed system, as the phase separation intensified with the increasing concentration of protein; this was similar what occurs with traditional polysaccharide-protein mixed gels [17,29]. Studies have identified three typical network types involved in protein and polysaccharide mixed gels: interpenetrating networks, coupled networks and phase-separated networks [3,4]. However, the networks in protein-polysaccharide mixed gels are usually heterogeneous, which may be the result of the interaction or entanglement of the aforementioned network structures. Chen et al. [4] also reported that both interpenetrating and interconnected networks existed in a sugar beet pectin-SPI DN gel induced by heating and laccase, in which an interconnected network was formed by covalent crosslinking of ferulic acid on SBP and tyrosine on SPI through laccase oxidation. Meanwhile, phase separation behavior was also observed in the CLSM image of this DN gel; this was regulated by the protein concentration. In addition, Hou et al. [3] reported that the highly hierarchical structure of a sugar beet pectin-soy protein DN gel was caused by the self-assembly of the two polymers, driven by phase separation behavior during the gelation process. Therefore, the formation of a CFG-SPI hydrogel network can be attributed to the combined effect of covalent crosslinking of polysaccharide molecules, protein thermal aggregation, covalent crosslinking between CFG and SPI, and phase separation. Furthermore, studies have indicated that the structures of mixed gels depend on the balance between the gelation process and phase separation [15]. Similarly, in our experiment, the microstructure of CFG-SPI DN hydrogel might depend both on the balance between the formation speed of the first network and the phase separation.

CLSM observations of DN hydrogels prepared with a constant CFG concentration of 2% and various SPI concentrations, i.e., 8%, 10%, and 13%, are shown in Figure 1g–i. It can be seen that when the SPI concentration was 8%, although a separate network formed, the concentration was so low that CFG phase was still dominant and continuous. With the increase of the SPI concentration, the microstructure of the DN hydrogels was converted to a bicontinuous pattern. The results imply that the gelation process of the DN hydrogel was accompanied by the occurrence of phase separation behavior, and moreover, that the competitive effect between these two actions led to the formation of the final DN hydrogel structure, which could be modulated by controlling the concentrations of protein and polysaccharide.

#### 3.1.2. Textural Properties

**Figure 2:**
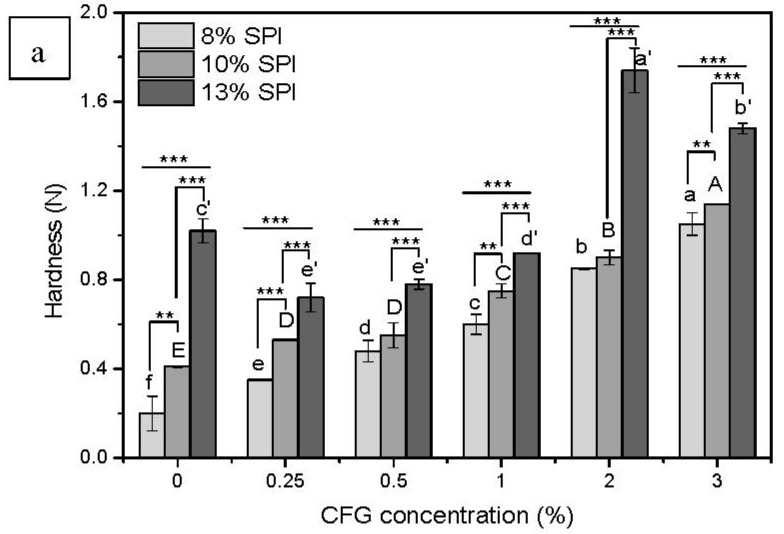

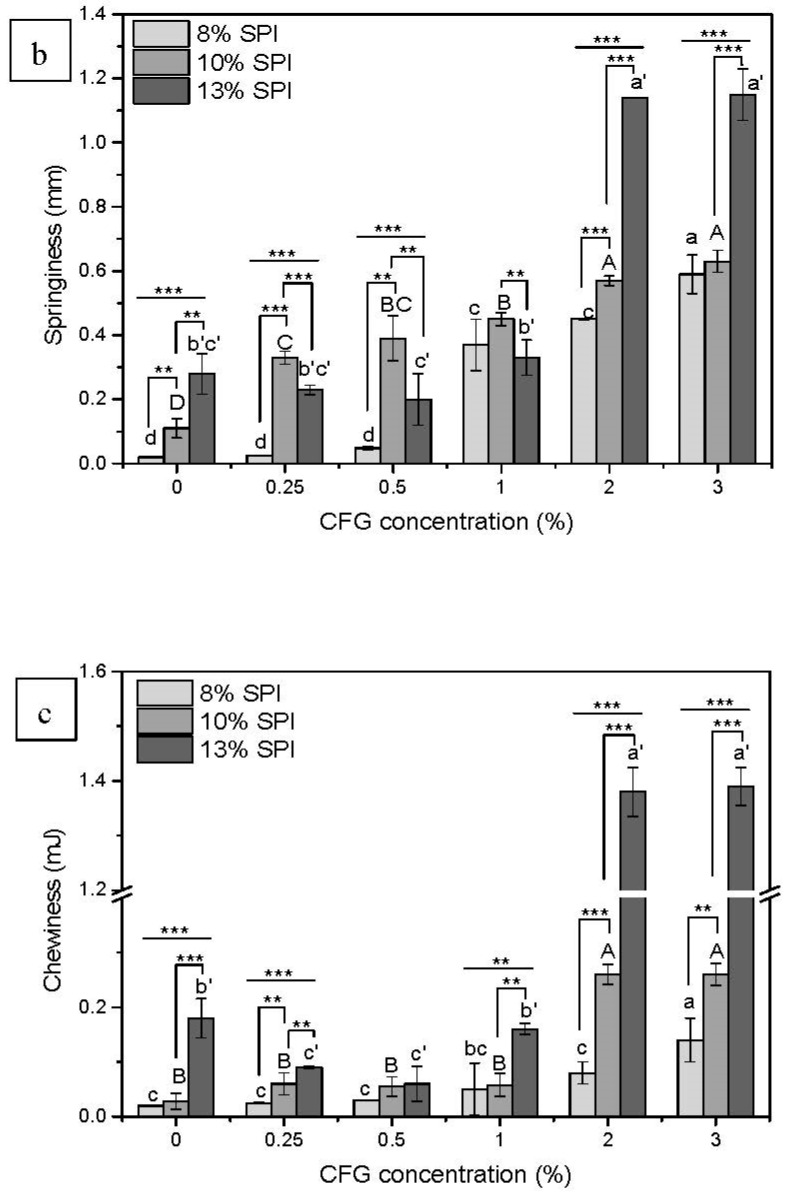
Textual properties of CFG-SPI DN hydrogels with various CFG/SPI proportions ((**a**) Hardness; (**b**) Springiness; (**c**) Chewiness, the concentrations of CFG and SPI were 0%, 0.25%, 0.5%, 1%, 2%, 3% and 8%, 10%, 13% (*w*/*v*), respectively). Different letters (a–f, A–E, a′–e′) indicate statistically significant differences between different CFG concentrations with SPI at a fixed concentration (8%, 10%, 13%, respectively) (*p* < 0.05). A different number of * indicates that the mean values between the two columns differ significantly, i.e., *, **, *** indicate *p* < 0.1, *p* < 0.05, *p* < 0.001, respectively.

The texture characteristics of DN hydrogels with different concentrations of SPI and CFG are exhibited in Figure 2. First, the effect of CFG concentration on the mechanical properties of the DN hydrogels was studied. For fixed SPI concentrations of 8% or 10% (*w*/*v*), as the CFG concentration increased, the hardness, springiness and chewiness of the DN hydrogel increased, surpassing those of the SPI single-network hydrogel. Notably, when the CFG concentration reached 2% (*w*/*v*), the enhancement of mechanical strength of this DN hydrogel was extremely significant. In previous research, we revealed the gelation mechanism of CFG-SPI DN hydrogel, that is, CFG gel served as the first stiff network skeleton and SPI gel then formed the second elastic and flexible network to fill in the first layer of the polysaccharide network; consequently, a DN hydrogel with high hardness and elasticity was obtained [11]. The increase in polysaccharide concentration in the mixed system facilitated the covalent cross-linking of ferulic acids between polysaccharide molecules, which increased the strength of the first polysaccharide hydrogel network, and ultimately led to the enhancement of the mechanical properties. However, interestingly, when the SPI concentration increased to 13% (*w*/*v*), although the hardness and springiness of the DN hydrogel showed a gradual increase with the increase in the amount of CFG added, when the CFG concentration was low (0.25%, 0.5%, 1%), the texture parameters of the prepared DN hydrogels were all lower than those of the SPI hydrogel (without the addition of CFG). This may have been due to the fact that a small amount of polysaccharide is prone to inducing a strong phase separation (greater than gelation at this time) under excessively high protein concentrations, and moreover, these weak polysaccharide hydrogel networks cannot effectively support the DN hydrogel but instead affect the formation of the subsequent protein gel network by causing a space-occupying effect, thereby weakening the DN hydrogel [4,30]. While sufficient CFG (2% and 3%) addition promoted both gelation and phase separation, enhanced gelation of covalent cross-linking dominated the mixed system, thereby significantly improving the textural properties of the DN hydrogel as compared to a SPI single-network hydrogel. In summary, the results demonstrated that not all DN hydrogels had stronger mechanical properties than single-network hydrogels, but rather, that their properties were strongly correlated to the concentrations of both DN hydrogel components.

The influence of SPI concentration (8–13%, *w*/*v*) on the textural properties of DN hydrogels was also investigated (Figure 2). The hardness, springiness and chewiness of most DN hydrogels increased with increasing SPI concentration at a fixed CFG concentration, except for the DN hydrogels prepared at both a relatively high SPI concentration (13%, *w*/*v*) and low CFG concentrations (0.25%, 0.5%, 1%, *w*/*v*); in these cases, the springiness was slightly lower than that of the DN hydrogel prepared with the corresponding low SPI concentration (10%). This may be explained by the fact that in the mixed gel system of high-concentration protein and low-concentration polysaccharide, phase separation was stronger than gelation, and the space-occupying effect of the polysaccharides prevented the proteins from forming an elastic network, resulting in a reduction of hydrogel springiness. Overall, the DN hydrogels constructed with both high SPI (13%, *w*/*v*) and CFG (2% & 3%, *w*/*v*) concentrations exhibited excellent texture characteristics as compared to other DN hydrogels. Therefore, the texture and microstructure of the DN hydrogels can be effectively controlled by tuning the concentrations of polysaccharide and protein.

#### 3.1.3. Water-Holding Capacity

**Figure 3:**
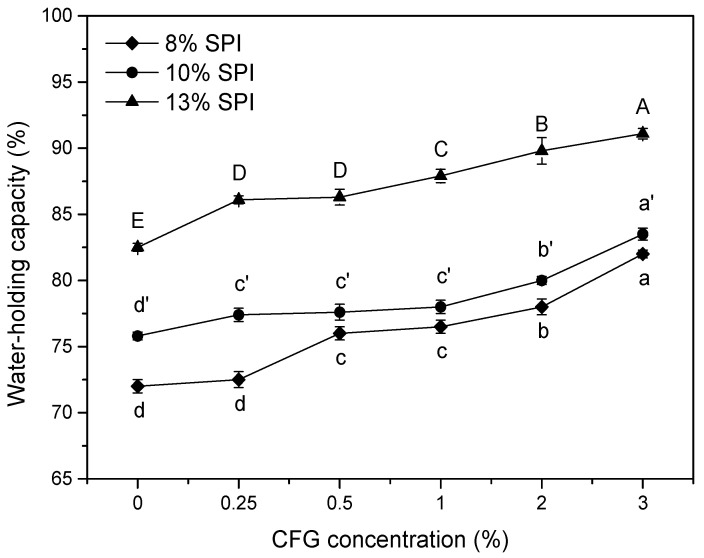
Water-holding capacity of CFG-SPI DN hydrogels with various CFG/SPI proportions (the concentrations of CFG and SPI were 0%, 0.25%, 0.5%, 1%, 2%, 3% and 8%, 10%, 13%(*w*/*v*), respectively). Different letters (a–d, a′–d′, A–E) indicate statistically significant differences between different CFG concentrations when SPI is at a fixed concentration (8%, 10%, 13%, respectively).

As shown in Figure 3, the WHC of the CFG-SPI DN hydrogels apparently changed with concentrations of CFG and SPI. As the CFG concentrations increased, the improvement of WHC was significant, which was consistent with the results shown in many studies on polysaccharide-protein mixed gels. A possible reason for this was that more covalent cross-linked networks formed gradually with the increasing concentration of CFG. According to our previous study [11], compared with single-network hydrogels, CFG-SPI DN had a more regular and denser network structure, in which some small holes were distributed among big holes due to the formation of interconnected and intertwined polysaccharide/protein double networks. This network structure allowed the DN hydrogel to trap more water which could not be removed under the currently applied centrifugation conditions. On the other hand, since the polysaccharide contained a large number of hydrophilic groups, its hydrogen bonding interaction with the water molecules also helped to enhance its ability to bind water [7].

Figure 3 also reveals a continuous improvement in WHC, along with an increase of SPI concentration with a constant CFG content, especially when the SPI concentration rose to 13% (*w*/*v*). The formation of the protein hydrogel network was the result of the balance between protein–protein, protein–solvent interactions, as well as the gravitation and repulsion between peptide chains. The high protein concentration facilitated the formation of more hydrogen bonds with water molecules, and thus, more water could be trapped [4]. In addition, unlike the coarse and irregular large-mesh structure of DN hydrogel prepared with a low protein concentration, the DN hydrogel with a high protein content was smoother and denser, and the capillary siphon effect was so significant that more water was retained.

### 3.2. Effect of pH Values on Properties of CFG-SPI DN Hydrogels

#### 3.2.1. Microstructure

**Figure 4:**
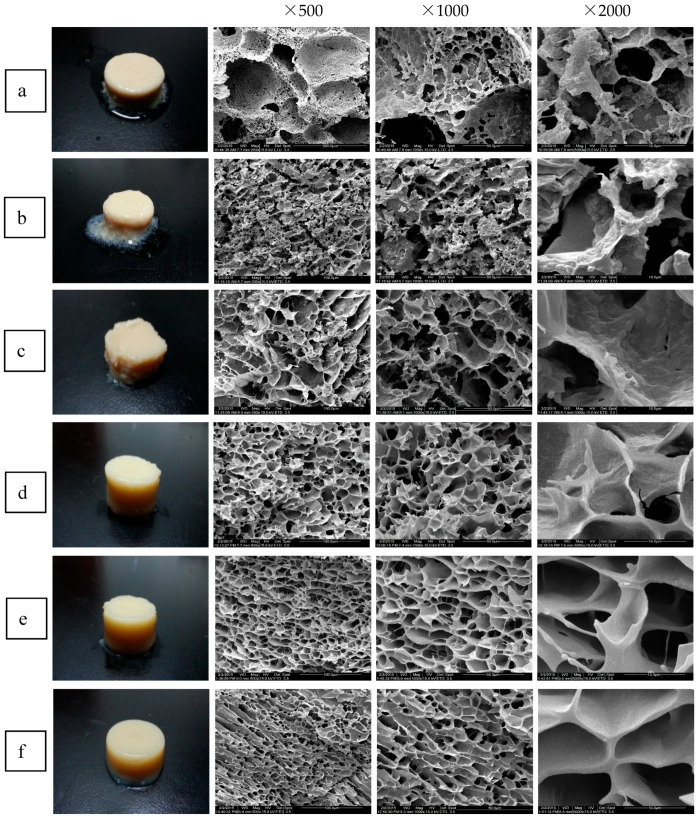
Appearance images and SEM observation of CFG-SPI DN hydrogels at different pH values. ((**a**) pH = 5; (**b**) pH = 5.5; (**c**) pH = 6; (**d**) pH = 6.5; (**e**) pH = 7; (**f**) pH = 7.5).

Images and SEM micrographs of CFG-SPI DN hydrogels at various pH values are shown in Figure 4. The DN hydrogel at pH 5.0 was white and coarse; it can be observed this hydrogel lost a great deal of water during formation, which might have been due to the relatively rapid formation rate of the hydrogel. Previous studies reported that the optimal pH value of laccase from white-rot fungus was 3.5–4.5, and that the enzyme activity was stable at pH 5.0–7.0 [31]. Additionally, it is well known that the isoelectric point (pI) of SPI is approximately at 4.6. These two factors led to the rapid formation of DN hydrogel at pH 5.0. With increasing pH (pH > 6), the DN hydrogels became more hydrated with less water separated while remaining more intact.

From the SEM images (Figure 4) of hydrogels at pH ≤ 6.0, an uneven hole structure may be observed at a magnification of 500 times, while at higher magnification, these hydrogels exhibited more irregular holes, and the pore walls were rough with some protrusions. As the pH increased, the microstructure of the DN hydrogels changed from disordered accumulation to a regular, honeycomb, three-dimensional network structure. When the pH reached 7.0 and 7.5, the pore diameter of the DN hydrogels became more uniformed and the pore wall was smoother and thicker. Changes in pH will affect the ionization and the net charge of protein molecules, thereby impacting the electrostatic attraction or repulsion of protein molecules, the binding ability of protein and water molecules, and the interactions between protein and polysaccharide molecules, which, in turn, affect the formation and maintenance of the hydrogel networks [20,32]. Studies have indicated that during protein thermal gelation, the relative speed of denaturation and aggregation determines the structure as well as the physical and chemical properties of hydrogels [33,34]. When the speed of aggregation is slower than that of unfolding, the denatured chains of protein will have a better orientation, which is conductive to the formation of a more regular network, while disorder and coarse structures will occur when aggregation takes place more rapidly. In this present study, when the pH value was close to the pI of SPI (Figure 4a,b), smaller electrostatic repulsion and stronger hydrophobic interaction allowed the proteins to aggregate quickly before unfolding, which resulted in the formation of larger protein aggregate particles, thereby forming a coarse but compact structure in which the pore sizes were varied and the pore walls were thin and brittle. Conversely, when the pH was distant from the pI, enhanced electrostatic repulsion allowed the proteins to fully unfold but slowly aggregate during heating, forming regular and uniform microstructures with smooth and thick pore walls [34]. On the other hand, under acidic conditions, the surface charges of the anionic polysaccharide CFG and SPI molecules were neutralized, resulting in weakened intermolecular interactions and forming a weak and rough network structure. However, at neutral pH, the increase in the net charges on the surface of the two polymers enhanced interactions which contributed to the formation of a more stable and regular network [33]. The above results indicate that pH has a great influence on the microstructures of DN hydrogels, and that adjusting the pH to neutral may alter the characteristics of the networks, i.e., from rough and disordered to regular and ordered.

#### 3.2.2. Textural Properties

**Figure 5:**
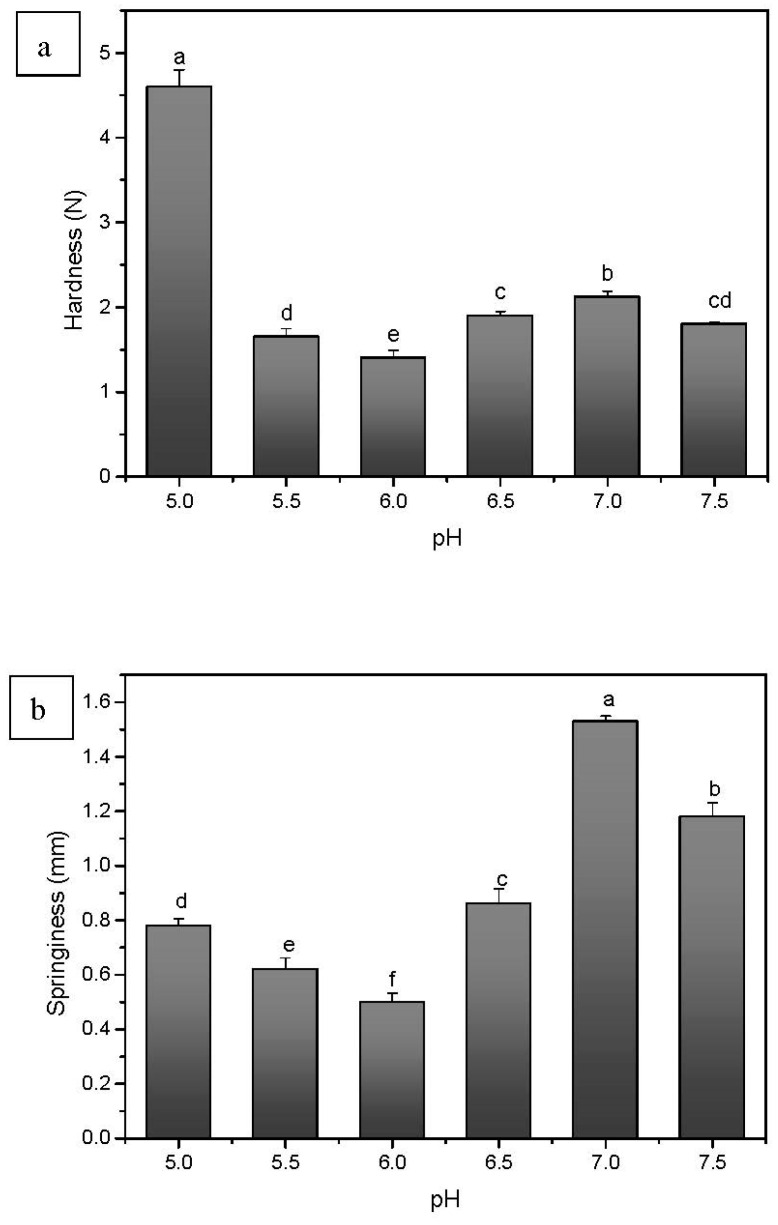

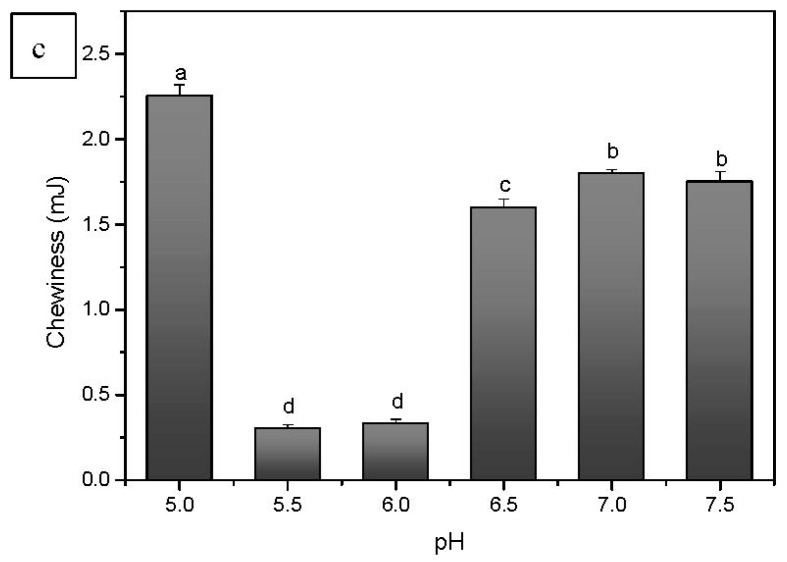
Textural properties of CFG-SPI DN hydrogels at pH 5.0–7.5. ((**a**) Hardness; (**b**) Springiness; (**c**) Chewiness). Different letters (a–f) indicate statistically significant differences between different pH values (*p* < 0.05).

The textural properties of CFG-SPI DN hydrogels at different pH values, i.e., from 5.0 to 7.5, are shown in Figure 5. These data suggest that with increasing pH values, the hardness of DN hydrogels decreases first and then increases. The DN hydrogel at pH 5.0 had the highest hardness (4.6 N), i.e., significantly higher than that at other pH values. This might have been because the rapid and disordered aggregation of protein molecules at a pH near the pI of SPI caused the hydrogel structure to be rough. At the same time, due to the dehydration and shrinkage of this hydrogel, the structure was more compact, resulting in a hard but poorly elastic hydrogel. As the pH gradually increased away from the pI, the aggregation rate of protein molecules slowed and the hydrogel network tended to be regular and ordered. Although the pore size became larger, the pore wall thickened, which enhanced hydrogel toughness. It was found that the springiness of the hydrogel at pH 7.0 reached 1.53 mm, which was about twice as high as that at pH 5.0 (Figure 5). Renkema et al. [35] found that soy protein formed more fine-stranded gels at pH 7.6, characterized by low strength and smooth appearance, while at pH 3.8, coarse gels were obtained with the high stiffness and granulated appearance. Sittikijyothin et al. [32] demonstrated that β-lactoglobulin-tara gum mixed gels prepared at pH 4.6 (the pI of the protein) were stronger and showed higher sensitivity to strain and larger clusters in comparison to similar gels prepared at pH 7.0. In addition, the formation of a polysaccharide-protein insoluble complex at the pI was the likely cause of the rough structure and poor elasticity of the DN hydrogels [36]. As for chewiness, the variation trend was similar to that of hardness. In summary, the hardness, springiness and chewiness were greatly influenced by pH values, and the various texture characteristics of the DN hydrogels obtained at different pH values were closely related to the microstructures. At pH 5.0, the DN hydrogel had the highest hardness and chewiness but poor elasticity, whereas at pH 7.0, the hydrogel had the highest springiness but insufficient hardness. Therefore, various DN hydrogel products with different texture can be tailored according to consumer requirements.

#### 3.2.3. Water-Holding Capacity

**Figure 6:**
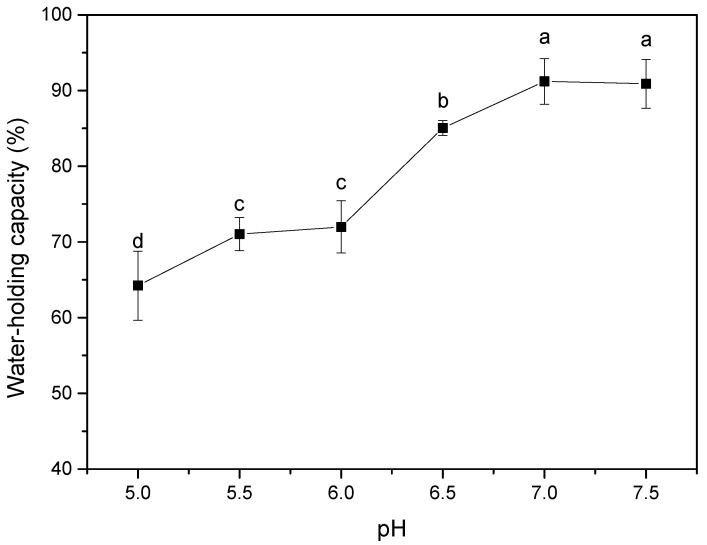
Water-holding capacity of CFG-SPI DN hydrogels at pH 5.0–7.5. Different letters (a–d) indicate statistically significant differences between different pH values (*p* < 0.05).

As shown in Figure 6, the WHC of the CFG-SPI DN hydrogels increased from 64.23% to 91.21% with increasing pH. As shown in the SEM micrograph of the DN hydrogels (Figure 4), the poor WHC of the DN hydrogels obtained at the low pH value (pH ≤ 6) might have been due the fact that the microstructures of these hydrogels were coarse and irregular, and a large amount of water was lost during the gelation process. As the pH increased to 7.0, the microstructure became smooth and regular, and holes were distributed uniformly and interconnected with each other, thereby allowing the hydrogel to retain more water [7]. Furthermore, the effect of pH on the intermolecular forces within the hydrogels can also explain the changes in WHC. Under acidic conditions, the net charges of SPI molecules and CFG molecules on the surface were reduced, so the electrostatic repulsions between the molecules were weakened and the hydrophobic interactions were enhanced. This facilitated interactions between the matrix molecules instead of with water, ultimately resulting in a decline in WHC [17,28]. With increasing pH, the increased net charges on the surface of the two molecules led to an increase in the electrostatic repulsion, which, in turn, promoted the interaction of matrix molecules and water molecules, greatly enhancing the WHC of the gel. In summary, the CFG-DN hydrogels prepared under neutral pH conditions had excellent WHC and springiness, as well as good hardness and chewiness.

## 4. Conclusions

In the present study, we demonstrated that the microstructures, textural properties and water-holding capacities of the CFG-SPI DN hydrogels can be influenced by the concentration of CFG/SPI and by pH. As the concentrations of CFG and SPI increased to 2% and 13% respectively, the DN hydrogel exhibited higher hardness, springiness, chewiness as well as WHC. In terms of the effect of pH (5.0–7.5), at pH ≤ 6.0, the microstructure demonstrated coarse and disordered accumulation, as observed by SEM, whereas with increasing pH values, the DN hydrogel showed a typical honeycomb network with smooth and thick pore walls. Although the DN hydrogel prepared at pH 5.0 had the highest hardness, its poor springiness and WHC limited its application. Compared with this hydrogel, the DN hydrogel at pH 7.0 possessed excellent springiness, WHC and appropriate hardness, which widened its potential for utilization in food processing. This study provides a further theoretical supplement for the construction of protein-polysaccharide DN hydrogels. By controlling the concentrations of components and pH values, DN hydrogels can be fabricated with desired textural characteristics, making it possible to improve food texture or even control the release of nutrients.
